# Development and calibration of a novel positive mindset item bank to measure health-related quality of life (HRQoL) in Singapore

**DOI:** 10.1371/journal.pone.0220293

**Published:** 2019-07-31

**Authors:** Yu Heng Kwan, Elenore Judy Uy, Dianne Carrol Bautista, Xiaohui Xin, Yunshan Xiao, Geok Ling Lee, Mythily Subramaniam, Janhavi Ajit Vaingankar, Mei Fen Chan, Nisha Kumar, Yin Bun Cheung, Terrance Siang Jin Chua, Julian Thumboo

**Affiliations:** 1 Program in Health Services and Systems Research, Duke-NUS Medical School, Singapore, Singapore; 2 Department of Rheumatology & Immunology, Singapore General Hospital, Singapore, Singapore; 3 Singapore Clinical Research Institute, Singapore, Singapore; 4 Centre for Quantitative Medicine, Duke-NUS Medical School, Singapore, Singapore; 5 Academic Clinical Programme for Medicine, Singapore General Hospital, Singapore, Singapore; 6 Department of Social Work, Faculty of Arts and Social Sciences, National University of Singapore, Singapore, Singapore; 7 Research Department, Institute of Mental Health, Singapore, Singapore; 8 Neuroscience and Mental Health, Lee Kong Chian School of Medicine, Singapore, Singapore; 9 Health Promotion Board, Singapore, Singapore; 10 Tampere Center for Child Health Research, University of Tampere and Tampere University Hospital, Tampere, Finland; 11 Department of Cardiology, National Heart Centre Singapore, Singapore, Singapore; 12 Office of Clinical, Academic & Faculty Affairs, Duke-NUS Medical School, Singapore, Singapore; 13 Yong Loo Lin School of Medicine, National University of Singapore, Singapore, Singapore; Leiden University Medical Center, NETHERLANDS

## Abstract

**Background:**

Positive mindset (PM) is an important domain of health-related quality of life in Singapore, a multi-ethnic urban city state in Southeast Asia. We therefore developed and calibrated a novel item bank to measure and improve PM.

**Methods:**

We developed an initial candidate pool of 48 items from focus groups, in-depth interviews and existing instruments locally developed and validated for use in Singapore. We administered all items in English to a multi-stage sample stratified for age and gender, of subjects with and without medical conditions recruited from the community and a hospital, and calibrated their responses using Samejima’s Graded Response Model. We evaluated a final 36-item bank with respect to Item Response Theory (IRT) model assumptions, model fit, differential item functioning (DIF), concurrent and known-groups validity.

**Results:**

Among 493 participants (49.3% male, 41.6% above 50 years old, 33% Chinese, Malay and Indian), bifactor model analyses supported unidimensionality: explained common variance of the general factor was 0.86 and omega hierarchical was 0.97. Local independence was deemed acceptable: the average absolute residual correlations were <0.06 and 3.3% of the total item-pair residuals were flagged for local dependence. The overall model fit was adequate and provided good coverage of the PM construct (theta range: -3.6 to +2.4). Five items exhibited DIF with respect to ethnicity and gender, but were retained without modification of scores because they measured important aspects of PM. Scores correlated in the hypothesized direction with a self-reported measure of global health (Spearman’s rho = -0.28, p<0.001) and discriminated between groups of participants with and without a self-reported diagnosis of a mood disorder (p = 0.007) adjusting for age, gender, ethnicity, education and marital status.

**Conclusion:**

The 36-item PM item bank demonstrated satisfactory psychometric properties for the English-speaking Singaporean population. IRT model assumptions were sufficiently met and scores showed concurrent and known-groups validity. Future studies to evaluate the validity of PM scores when items are administered adaptively are needed.

## Introduction

The World Health Organization (WHO) states that health is a state of complete physical, mental and social well-being, and not merely the absence of disease or infirmity.[1] PM is defined as thinking positively in life.[2] Although static instruments have been developed, we were not able to identify an item bank specifically measuring this latent construct. [3, 4]

Item banks that have been developed were initially focusing largely on latent constructs related to physical traits or functions.[5] Item banks that measure psychological constructs such as resilience and emotional distress have also been developed recently.[6, 7] In Singapore, the Positive Mental Health Instrument (PMHI) and the Singapore Mental Wellbeing (SMWEB) scales have been developed.[3, 4] Although similar latent constructs were being measured, PMHI and SMWEB were conceptualized as multidimensional constructs encompassing positive affect, satisfaction, and psychological functioning. In contrast PM refers to the amount of optimism one has.[3] Being able to measure the magnitude of how an individual thinks positively in life will allow interventions to be created and reduce the negative impact of poor PM.[8] Also, with the high prevalence of mental health conditions, the ability to maintain a PM may reduce the number of patients with mental health conditions.[9] The development of a PM item bank is a foundation for measuring PM and will enable development of short static instruments or computer adaptive testing (CAT) to measure PM as a latent construct.

Further, despite the popularity of HRQoL instruments such as the World Health Organisation Quality of Life Scale (WHOQOL-BREF) and Short Form-36 Health Surveys (SF-36), most of these instruments have been developed and used in Western populations and adapted later for use in other populations. Hence, to address the above gaps, we developed a comprehensive and culturally sensitive PM item bank to measure PM in Singapore. The aim of this study was to calibrate an item bank of PM that includes important and culturally appropriate items measuring PM that can be used across different age, gender and ethnic groups. A successfully calibrated item bank will allow us to be able to develop CAT or short static instruments to measure PM with accuracy and precision in Singapore.

## Methods

This institutional board review-approved study (Ref 2014/916/A) consisted of the following sequential steps: development of a candidate item bank, administration of candidate item bank via a community and hospital-based survey, and item bank calibration through assessing the assumptions of item response theory (IRT), fitting the responses to IRT model, testing for differential item functioning (DIF) and testing the PM scores of the item bank using a priori hypotheses.

### Development of a candidate item bank

The detailed methodology for the development of candidate items has been reported separately.[2, 10–12] In brief, we adapted the Patient Reported Outcome Measurement Information System (PROMIS) Qualitative Item Review (QIR) protocol[13], with input and endorsement from expert panels (comprised patients, members of the general public, and experts in psychology, social work and psychometrics). Items were generated from thematic analyses from focus groups and in-depth interviews and a literature search to identify studies that developed or validated a health-related quality of life instrument among adults in Singapore. Item from these sources were “binned” and “winnowed” (as detailed in the PROMIS QIR protocol) by two independent reviewers, blinded to the source of the items, who harmonized their selections to generate a list of candidate items (each item representing a subdomain). An expert panel reviewed and refined the face and content validity of these candidate items.

### A community and hospital-based survey

We recruited Singapore citizens or permanent residents from the community and from the Singapore General Hospital Campus. We sampled 75% English and 25% Chinese (Mandarin) speaking participants separately. Within each language sampling frame, a purposive sample of participants was drawn based on age, gender, ethnicity and presence or absence of chronic illnesses. The list of chronic illnesses was based on the Singapore Burden of Disease Study [14] and is detailed in S1 Table. The presence or absence of a chronic illness was based on a participant’s self-report of having been diagnosed of an illness by a physician. Participants were categorized into well, mildly unwell and unwell, according to number and severity of chronic illnesses. We excluded individuals who had impairments that precluded a meaningful exchange of ideas or other conditions that prohibited them from carrying out a normal interview, such as severe mental illness and cognitive impairment. In order to include participants with a wide spectrum of health, we predefined the proportion of participant recruitment in health categories to be 35% well, 15% mildly unwell, and 50% unwell.

Participants from the community were sampled using a residential household sampling frame of public housing, which 82% of Singaporeans reside in[15]. The primary sampling units were plots of land with approximately equal numbers of households, stratified according to geographic location and dwelling type. Households in each primary sampling unit were selected based on fixed route rules and skip patterns based on pre-specified ethnic and age quotas. Only one respondent per household was selected for a face-to-face interview. Three call attempts to each household were made at different times of the day with at least 1 visit on a non-work day (Saturday or Sunday). This residential household based sampling method has been used in the Singapore National Health Survey since 2004 [16, 17]. The response rate of the study was computed using the standard set by the Council of American Survey Research Organization [18], generally defined as the number of completed Interviews divided by the number of eligible reporting units in sample. We engaged Nielsen Research Company to conduct the standardized surveys on behalf of the study team.

Interviewers administered the items developed from a previous study conducted by our team in English.[10, 11, 19] Interviewer administration was selected so that illiterate subjects (who form 20% of Singapore population) could be included so that the resulting item bank could be applied to the entire English speaking population in Singapore (Test administration for illiterate subjects could be accomplished through the use of interviewer- or technology-assisted formats).[20] There were 48 items presented to the participants with 5-level item response options adapted from the PROMIS. The response options of the item were “Never”, “Seldom”, “Sometimes”, “Usually” and “Always” for items on frequency and “Not at all”, “Mildly”, “Moderately”, “Quite a lot” and “Extremely” for items on intensity. We collected demographics including age, gender, ethnicity, education and current marital status. We collected a single-item, participant-reported assessment of global health for comparison.

### Item bank calibration

We adapted the methodology published by PROMIS to calibrate the English version of the PM item bank. To test IRT model assumptions, we evaluated unidimensionality using factor analyses, which included Exploratory (EFA) and Confirmatory (CFA) and Exploratory bifactor analyses (with orthogonal rotation). We reported the latter if EFA and CFA showed evidence for secondary dimensions. In the exploratory bifactor analyses, we fit models with two, three and four group factors to clarify any underlying secondary dimensions. After ascertaining adequacy via conventional fit criteria, we used the average relative parameter bias (ARPB), explained common variance (ECV) of the general factor and omega hierarchical (omegaH) to assess whether the presence of multidimensionality does not disqualify interpretation of the instrument as being primarily unidimensional. To calculate these bifactor indices, we used a Microsoft Excel based calculator[21]. We checked for monotonicity using individual category response curves. We evaluated local independence by examining the residual correlation matrix from the single factor CFA. The specific criteria we used are given in Table 1. We used Mplus Version 8.0 software to check for unidimensionality and local independence[22]. We fitted Samejima’s graded response model (GRM), a non-Rasch model, to calibrate the items and estimated parameters via marginal maximum likelihood using the Xcalibre 4.2 IRT software (Assessment Systems Corporation, USA). We tested adequacy of overall model fit as well as individual item fits using a chi-square fit statistic. We checked for DIF by these subgroups: age (age < 50 versus age ≥50), gender (Male/Female) and ethnicity (Chinese vs non-Chinese), using likelihood chi-square statistics from ordinal logistic regression, comparing models with and without subgroup membership as predictor. We tested for uniform and non-uniform DIF using a specially written syntax in IBM Statistics Version 23.0 (http://www-01.ibm.com/support/docview.wss?uid=swg21572191, downloaded on 18 December 2017). We assessed items for concurrent validity with a self-reported measure of global health (“In general, would you say your health is: Excellent, Very Good, Good, Fair or Poor?”, hypothesizing a moderate negative correlation (Spearman’s rho < -0.25) between PM theta scores and the global health self-report. A negative correlation was hypothesized with a higher PM score indicating a more PM and a lower score on global health indicating better health. We also assessed known-groups validity using Analysis of Variance (ANOVA), hypothesizing that PM scores could discriminate between participants with and without a self-reported physician diagnosis of anxiety or depression and those who did not, adjusted for participant’s age (20–35, 36–49, 50 and above), gender (Male/Female), completion of secondary education (Yes/No) and current marital status (Single, Married, Divorced/Widowed/Separated) as covariates. We used a 5% significance level. Concurrent and known-groups validity were carried out using the IBM Statistics Version 25.0 software.

**Table 1:** Criteria for evaluating Item Response Theory assumptions and results.

**Unidimensionality**				
Approach	Criterion	Reference	Results	Criterion met?
Exploratory factor analysis	Percentage of variance accounted for by first factor > 20%	PROMIS^16^	20.9%	Yes
	Ratio of first to second eigenvalues > 4.0	PROMIS^16^	11.0	Yes
Confirmatory factor analysis	Comparative fit index >0.95	PROMIS^16^	0.882	No
	Tucker-Lewis Index > 0.95	PROMIS^16^	0.875	No
	Root mean square error of approximation < 0.06	PROMIS^16^	0.116	No
	Standardised root mean residual< 0.08	PROMIS^16^	0.079	Yes
Bifactor analyses	ARPB < 10%	Muthén, Kaplan, and Hollis (1987)[37]	4.6^§^	Yes
	General ECV > 0.70	Reise, Bonifay and Haviland (2013) ^17^	0.814^ǂ^	Yes
	OmegaH > 0.80		0.969^¥^	Yes
	General ECV > 0.60 and OmegaH>0.70	Reise, Schienes, Widaman and Haviland (2013) ^15^		Yes
	**Local Independence**				
Residual correlation matrix	Average absolute residual correlations < 0.10	PROMIS^16^	0.059	Yes
	Percentage of residual correlations above 0.20	Artmann et al 2010^18^	3.3% (21 of 630)	No threshold given

Abbreviations: Average relative parameter bias (ARPB), Explained common variance (ECV), item explained common variance (IECV), omega Hierachical (OmegaH).

^§^Maximum ARPB among three exploratory bifactor models with 2,3 and 4 group factors. See Table 3.

^ǂ^Minimum general factor ECV attained among three exploratory bifactor models with 2, 3 and 4 specific factors. See Table 3.

^¥^ Minimum OmegaH attained among three exploratory bifactor models. See Table 3.

## Results

Thirty-six of 48 items were retained in the final PM item bank after reviewing initial IRT model fits and adequacy checks and consulting with the expert panel. As this paper focuses on the calibration of the PM item bank, the detailed results of the item generation are being reported separately.[11]

A total of 676 subjects completed the PM item bank survey in English (n = 493) or Chinese (n = 183). As this paper focuses on the analysis of the English PM item bank, a total of 493 participants were analysed in this PM item bank calibration study. Characteristics of the study participants are shown in Table 2.

**Table 2:** Characteristics of study subjects.

	Frequency (%) N = 493
*Age*	
21–34 years old	120 (24.3)
35–49 years old	168 (34.1)
50 and above	205 (41.6)
*Gender*	
Male	243 (49.3)
Female	250 (50.7)
*Ethnic Group*	
Chinese	165 (33.5)
Malay	161 (32.7)
Indian	167 (33.9)
*Health*	
Well	180 (36.5)
Mild unwell	62 (12.6)
Unwell	251 (50.9)
*Marital status*	
Single	127 (25.8)
Married	338 (68.5)
Separated/divorced/widowed	28 (5.7)
*Completion of secondary education (10 years of education)*	
Yes	397 (80.5)
No	96 (19.5)

### Item analyses

**Fig 1:**
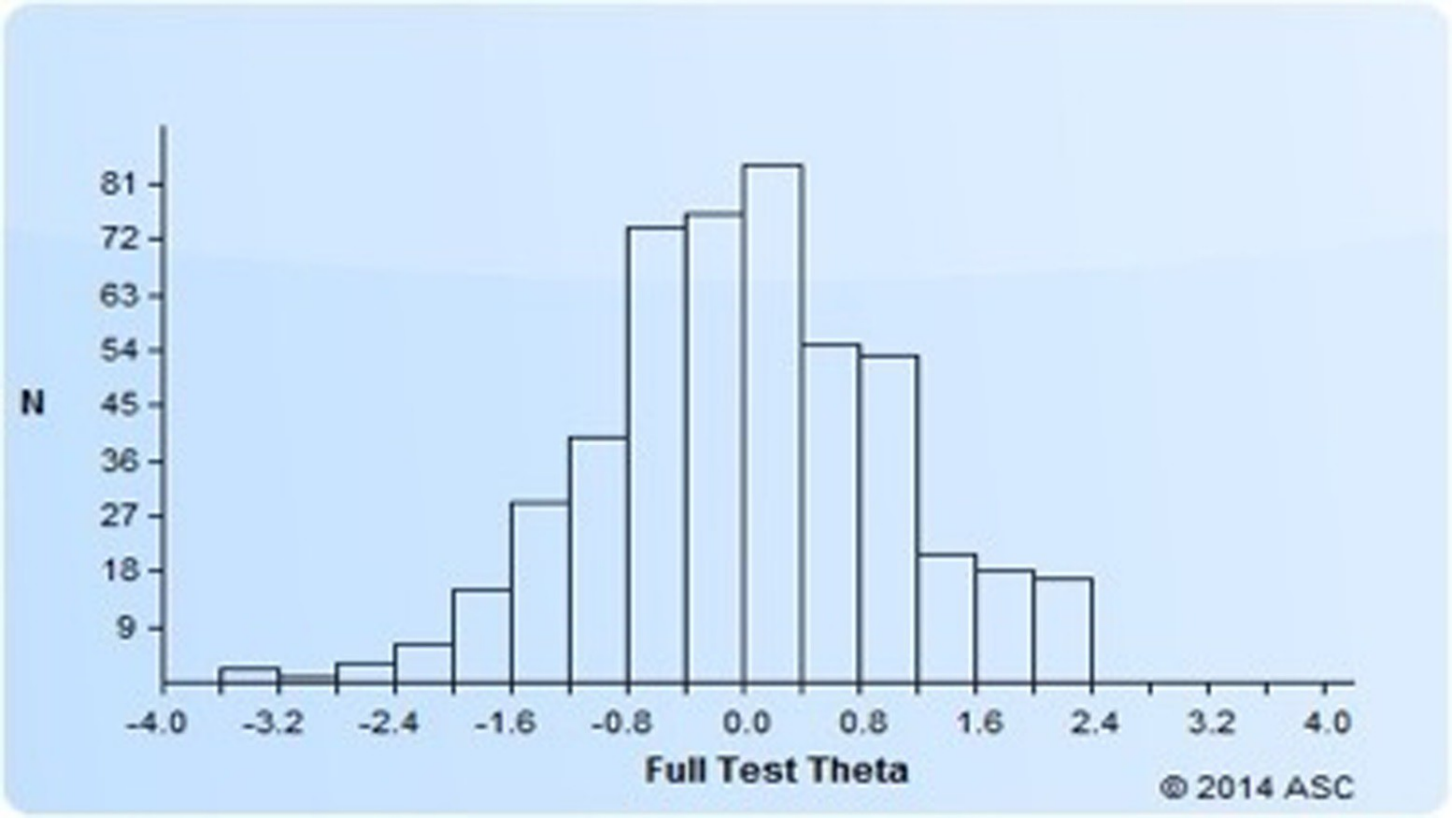
Theta estimates for all calibrated items.

Cronbach’s alpha was 0.97, indicating very high inter-item consistency. The mean item-to-total score correlation was 0.68 (SD = 0.08). Correlations ranged from 0.47 to 0.78. Item means ranged from 3.16 to 4.49. The percentage of non-response at the item level was practically nil, ranging from zero to at most 0.4%. As shown in Fig 1, there is good coverage of the PM construct (theta range: -3.6 to +2.4).

### IRT Assumptions of unidimensionality, local independence and monotonicity

Unidimensionality was assessed using EFA, CFA and bifactor analyses. In the EFA, 20.9% of the variance was explained by the first factor. The ratio of the first and second highest eigen value was 11.0 (Table 1). Both findings met recommended criteria for assessing unidimensionality. However, CFA showed Comparative Fit Index (CFI) <0.95, Tucker-Lewis Index (TLI) <0.95 and Root Mean Square Error of Approximation (RMSEA)>0.06, which suggested the existence of secondary dimensions (Table 1). Bifactor analyses showed that the level of disturbance in item parameter estimates attributable to multidimensionality was slight. Particularly, item loadings on the single-factor CFA and item loadings on the general factor of the bifactor models were not different according to the average relative parameter bias which were all under 5% (Table 3). Moreover, across all bifactor models, the attained ECVs and omegaHs of the general factor were above 0.80 and 0.95 respectively, much higher than Reise et al’s suggested criteria (ECV>0.6 and omegaH>0.7) [23]. Consequently, the instrument can be interpreted as being primarily unidimensional despite the presence of some multidimensionality. Examination of the residual correlation matrix indicated little local dependence: the average value of the residual correlations was <0.06 which was less than the 0.1 threshold. The proportion of item-pairs having problematic residual correlations (i.e., greater than 0.20) was 3.3% (21 of 630). Items 25 and 26 which covered religion and spirituality accounted for 12 out of the 21 problematic residual correlations. We appraised the extent of local dependency to be minor as not to compromise the accuracy of IRT parameter estimation. In terms of monotonicity, we found that none of the items departed from monotonicity in terms of improper ordering.

**Table 3:** Summary of results of bifactor analyses.

Item ID	Item	Single-factor CFA loadings	Exploratory Bifactor analysis general factor loadings		
			2 group factors	3-group factors	4-group factors
MQ01	I am able to accept people as they are	0.660	0.660	0.650	0.657
MQ15	Overall, I am satisfied with my life	0.780	0.784	0.777	0.784
MQ22	I feel enthusiastic	0.805	0.809	0.793	0.804
MQ29	I try to see the lighter side of stressful situations	0.759	0.763	0.747	0.755
MQ02	I am able to accept the way things work out	0.784	0.790	0.779	0.790
MQ09	I am a cheerful person	0.740	0.746	0.731	0.748
MQ11	I am able to bounce back after setbacks	0.768	0.776	0.768	0.774
MQ16	I am able to see the good in everyone	0.792	0.803	0.791	0.807
MQ31	I am able to relax	0.806	0.815	0.806	0.815
MQ03	I am able to accept who I am	0.787	0.793	0.788	0.798
MQ17	I am able to handle my negative feelings	0.789	0.798	0.792	0.794
MQ23	I feel enthusiastic about life	0.856	0.865	0.856	0.860
MQ24	I enjoy life	0.861	0.870	0.862	0.869
MQ18	I am able to manage my anger	0.717	0.728	0.728	0.734
MQ25	I find comfort in my religion or spiritual beliefs	0.756	0.549	0.550	0.545
MQ30	I am able to see things positively	0.830	0.833	0.836	0.843
MQ32	Overall, I feel confident	0.836	0.839	0.843	0.849
MQ12	I am able to cope with life's challenges	0.789	0.796	0.804	0.801
MQ19	I do not let my worries overwhelm me	0.802	0.807	0.811	0.810
MQ26	I find comfort in my religious beliefs	0.762	0.566	0.571	0.562
MQ33	I feel hopeful about the future	0.815	0.817	0.823	0.815
MQ06	I am able to appreciate each day fully	0.807	0.813	0.818	0.809
MQ13	I am able to deal with stress	0.778	0.785	0.794	0.785
MQ21	I feel in control of my life	0.808	0.813	0.819	0.809
MQ34	I am able to motivate myself	0.850	0.859	0.864	0.857
MQ07	I am able to appreciate what each day brings	0.789	0.797	0.803	0.783
MQ14	Overall, I feel contented with my life	0.741	0.752	0.760	0.738
MQ20	I am able to manage my worries	0.797	0.811	0.816	0.794
MQ28	I try to see the funny side of stressful situations	0.749	0.763	0.767	0.747
MQ41	In most ways my life is close to my ideal	0.638	0.597	0.591	0.595
MQ43	I feel at peace with myself	0.693	0.637	0.626	0.645
MQ45	I live a meaningful life	0.755	0.699	0.691	0.708
MQ47	I believe I am a good person	0.532	0.470	0.462	0.474
MQ42	I am clear about what I want in life	0.787	0.722	0.726	0.708
MQ44	I feel that my life has a purpose	0.751	0.667	0.672	0.652
MQ46	I take pride in my achievements	0.687	0.654	0.657	0.636
Bifactor analysis statistics				
Average relative parameter bias			4.4	1.5	4.6
ECV of general factor§			0.861	0.833	0.814
OmegaH			0.972	0.969	0.969
Maximum ECV of group factors			0.074	0.076	0.075

Abbreviations: Confirmatory factor analysis (CFA), explained common variance (ECV), omega hierarchical (omegaH).

§ Mean IECV is taken across the 3 exploratory bifactor models (2-group, 3-group and 4-group factors)

### IRT Calibration and Fit

PM items were scored so that higher scores indicated a more positive mindset. The overall fit of the GRM was found adequate (chi-square = 1616.3, df = 1952, p = 1.000). The items and parameter estimates are presented in Table 4. Using a significance value of 0.01, no item was found to misfit the GRM and p values ranged from 0.029 to 1.000 with a mean of 0.70. Item discrimination parameters varied from 0.68 to 1.72 (mean = 1.27, SD = 0.26) and item difficulty parameters ranged from -4.97 to 1.44. The latent PM trait covered by the items ranged from -3.6 to +2.4, showing more extensive coverage in the lower compared to higher PM traits. Test information was highest at latent trait scores between -3.5 and +0.5 with maximum attained at -2.90. At this range, the conditional standard error of measurement (CSEM) was less than 0.20 and at the maximum, it was 0.144. The CSEM was less than 0.31 (roughly, a reliability of 0.90) for scores below +1.5, and greater than 0.5 for scores above +2.0. Hence lower PM trait scores are measured with greater precision than higher PM trait scores.

**Table 4:** Item response theory calibration results.

Item ID	Mean	Item-total correlation	Cronbach’s alpha if deleted	IRT					GRM item fit p-value
				Discrimination	B1	B2	B3	B4	
MQ01	4.3	0.60	0.973	0.976	-4.53	-3.38	-1.59	0.22	0.928
MQ15	4.2	0.72	0.973	1.368	-3.56	-2.69	-1.25	0.28	0.672
MQ22	4.0	0.75	0.973	1.428	-3.46	-2.69	-1.25	0.28	0.065
MQ29	4.1	0.69	0.973	1.206	-3.45	-2.93	-0.99	0.46	0.379
MQ02	3.2	0.72	0.973	1.349	-3.32	-1.06	0.41		0.973
MQ09	3.2	0.67	0.973	1.149	-3.09	-1.17	0.32		0.667
MQ11	4.1	0.72	0.973	1.308	-3.69	-2.41	-1.06	0.59	0.985
MQ16	4.2	0.73	0.973	1.365	-3.43	-2.86	-1.19	0.40	0.989
MQ31	4.2	0.74	0.973	1.437	-3.48	-2.86	-1.19	0.40	0.914
MQ03	4.5	0.68	0.973	1.341	-4.03	-3.08	-1.72	-0.28	1.000
MQ17	4.2	0.72	0.973	1.373	-3.27	-2.58	-1.14	0.38	0.979
MQ23	4.1	0.78	0.972	1.629	-3.21	-2.28	-0.93	0.37	0.105
MQ24	4.3	0.77	0.973	1.722	-3.06	-2.32	-1.20	0.07	0.998
MQ18	4.1	0.67	0.973	1.111	-3.83	-2.87	-1.03	0.69	0.999
MQ25	4.3	0.47	0.974	0.758	-3.39	-2.96	-1.54	-0.13	0.946
MQ30	4.3	0.74	0.973	1.523	-3.37	-2.90	-1.29	0.21	1.000
MQ32	4.2	0.75	0.973	1.526	-3.39	-2.53	-1.18	0.21	0.999
MQ12	4.2	0.71	0.973	1.311	-3.61	-2.74	-1.24	0.40	0.892
MQ19	4.1	0.74	0.972	1.420	-3.20	-2.38	-0.97	0.37	0.026
MQ26	4.3	0.48	0.974	0.809	-3.25	-2.69	-1.50	-0.25	0.644
MQ33	4.3	0.73	0.973	1.529	-2.95	-2.52	-1.18	0.15	0.274
MQ06	4.3	0.73	0.972	1.466	-3.90	-2.64	-1.32	0.04	0.838
MQ13	4.1	0.70	0.973	1.307	-3.37	-2.59	-0.94	0.44	0.561
MQ21	4.2	0.73	0.973	1.418	-3.07	-2.48	-1.16	0.23	0.564
MQ34	4.3	0.76	0.973	1.690	-2.96	-2.40	-1.32	0.16	1.000
MQ07	4.3	0.69	0.973	1.420	-3.28	-2.81	-1.41	0.12	0.638
MQ14	4.2	0.66	0.973	1.186	-3.36	-2.69	-1.26	0.25	0.913
MQ20	4.1	0.72	0.973	1.409	-3.13	-2.37	-1.06	0.38	0.992
MQ28	4.0	0.68	0.973	1.214	-2.97	-2.41	-0.75	0.49	0.963
MQ41	3.6	0.59	0.973	0.844	-3.15	-2.12	-0.21	1.44	0.137
MQ43	4.0	0.62	0.973	0.959	-3.90	-2.56	-0.92	0.87	0.343
MQ45	4.0	0.67	0.973	1.136	-3.18	-2.48	-0.90	0.56	0.931
MQ47	4.2	0.48	0.974	0.681	-4.97	-3.78	-1.58	0.59	0.511
MQ42	4.1	0.69	0.973	1.195	-3.16	-2.56	-1.03	0.57	0.107
MQ44	4.1	0.64	0.973	1.015	-3.67	-2.56	-1.03	0.57	0.663
MQ46	3.9	0.63	0.973	0.980	-3.32	-2.30	-0.82	0.73	0.553

Abbreviation: Item response theory (IRT), graded response model (GRM), threshold value (b)

### Differential item function detection

At the 1% level of significance, none of the items were found to have significant age-related DIF. Five items were flagged for statistically significant DIF. Two items showed uniform DIF with respect to gender, MQ19 (“*I do not let my worries overwhelm me”)* and MQ07 (“*I am able to appreciate what each day brings*”). In the former item, men had greater odds of endorsing higher frequency options compared to women whereas in the latter, men had lower odds of endorsing higher frequency options than women.

Three items showed uniform DIF with respect to ethnicity, MQ25 (“*I find comfort in my religion or spiritual beliefs*”), MQ26 (“*I find comfort in my religious beliefs*”) and MQ13 (“*I am able to deal with stress*”). On the first two items, Malays and Indians (i.e., non-Chinese) showed greater odds of endorsing higher frequency options compared with the Chinese. In the third item on dealing with stress, Chinese had greater odds of choosing higher frequency options than Malays and Indians. Response options were ordered in frequency as Always, Usually, Sometimes, Seldom and Never and the response variable in the ordinal regression is the log odds of endorsing higher versus lower frequency options.

### Concurrent and known-groups validity evaluation

The Spearman correlation between PM scores and a self-report measure of global health was r = -0.28, supporting the hypothesis of a moderate correlation between the two measures and therefore demonstrating concurrent validity. After adjusting for age, gender, completion of secondary education and current marital status, a statistically significant mean difference was found in PM theta scores between participants with a self-reported diagnosis of anxiety or depression. The mean difference found was 0.62 (95% CI: 0.17 to 1.07 and p = 0.007), demonstrating known-groups validity. We categorized marital status as single, married or widowed/divorced/separated. We also explored categorizing marital status as currently married vs not currently married, and found the same results ie subjects with a diagnosed mood disorder had significantly lower mean PM scores than those without. This further supports robustness of this assessment of known-groups validity. The results of the validity evaluation are in Table 5.

**Table 5:** Evaluation of concurrent and known-groups validity.

**Self-reported global health** **(Concurrent validity)**	**N (%)**	**Positive Mindset theta score means (95% CI)**	**Spearman’s rho**
Excellent	28 (6.0)	0.424 (0.05 to 0.80)	- 0.28
Very Good	127 (26.0)	0.286 (0.09 to 0.48)	
Good	246 (50.0)	- 0.147 (-0.29 to 0.00)	
Fair	82 (17.0)	-0.438 (-0.66 to -0.21)	
Poor	10 (2.0)	-0.501 (-1.11 to 0.11)	
**Self-reported diagnosis of anxiety or depression** **(Known groups validity)**	**N (%)**	**Positive Mindset theta score means (95% CI)**	**Adjusted mean difference**^**#**^ **(95% CI)**
Yes	473 (96.0)	-0.67 (-1.11 to -0.22)	0.62(0.17 to 1.07), p = 0.007
No	19 (4.0)	-0.05 (-0.17 to 0.07)	

^#^adjusted for sex (male vs female), age group (21–34, 35–49, 50 and above), ethnicity (Chinese, Malay, Indian), completion of secondary education (Yes vs No) and current marital status (Married vs Single/Separated/Divorced)

Abbreviations: Positive mindset (PM)

## Discussion

This study described the calibration of a culturally sensitive item bank for PM for the Singapore population. Items from this PM item bank were derived from (1) extensive qualitative research to identify and incorporate perspectives from subjects in the population, representing a wide spectrum of healthy and ill subjects (with chronic diseases) (2) item from and involvement of investigators who developed static instruments measuring related concepts in the same population. The item bank we developed has high content validity in terms of relevance to the value of people in the right socio-cultural context, which can be generalizable to both healthy adults and those having chronic illnesses. The calibration processes aligned with the approach espoused by the PROMIS group [23–28]. The findings of this successful calibration indicate that this psychological item bank is a promising tool for measuring PM in the population.

PM is a novel construct and is of increasing importance to measure in order to improve optimism, which is an important construct with wide ranging positive impact on health. For example, high PM has been shown to reduce the incidence of mental health issues and may also ameliorate the impact of mental disease.[9] The PM item bank can be used in mindfulness based intervention trials in community or hospital based settings.[29] Also, the PM item bank potentially can be used to assess effectiveness of workplace-based programs to improve PM.[30] Given the potential application of the PM item bank, more research is needed to understand in depth the impact of PM on HRQoL, for examples in area of workplace stress and resillence building.[31]

The analyses of the IRT assumptions show that the required assumptions of unidimensionality and local independence are met. As we did not have prior expectation about the structure of any underlying dimensions, we ran exploratory rather than confirmatory bifactor analyses, positing models with two, three and four group factors. After ascertaining via conventional fit criteria that these models were adequate, we evaluated bifactor-specific indices which verified essential unidimensionality which we successfully did.[24] Both the GRM and DIF tests for gender and ethnicity flagged out five items. This represents less than 15% of the total number of items in the PM item bank. Although this is not ideal in calibration of item bank, other research groups have encountered a similar situation [32, 33]. The expert panel recommended retaining these items due to their important content validity and modest impact of the DIF [19, 33, 34]. In the future, these items may be removed or revised.

This study also supports the concurrent construct validity of the PM item bank. Our hypothesis testing showed good discriminative properties between participants with or without a reported diagnosis of anxiety and depression. Mental well-being comprised of various psychological constructs and overall physical well-being contributes to their mental well-being as well.[35] However, the number of participants with mood disorder is very small. Further tests are needed to assess the reproducibility of the results in a purposively sample of subjects with and without a diagnosis of a mood disorder.

We recognize several limitations of this study. First, a significant number of eligible subjects were excluded because quota for these subjects had been met. However, partly because of the use of quota sampling, the demographics in our sample are comparable to that of the population in Singapore.[36] Second, we only included 493 subjects in the analyses. However, our results fulfilled the needed assumptions of IRT calibration as set out by PROMIS. Third, the PM item bank may have poorer coverage on higher PM trait but better coverage on lower PM trait. However, this will unlikely be a problem if we use this item bank in clinical trials whereby patients of interest will likely have poorer PM than the general population.[3] Last, due to resource constraints and questionnaire fatigue, we only used self-reported measures of global assessment and self-reported diagnosis of depression and anxiety (rather than a psychological or mood scale) to measure concurrent validity and known-groups validity respectively. Future research should consider using a validated scale such as the Hospital Anxiety and Depression Scale to further evaluate the validity of the PM item bank.

In conclusion, we developed and calibrated a 36-item bank for PM that is relevant to the English-speaking Singaporean population and applicable to healthy adults and those having chronic illnesses. This would be promising item bank for the subsequent development of relevant short form or CAT to facilitate routine clinical use.

## Supporting information

S1 Table Chronic illness qualifying for patient recruitment. Based on the 2010 Singapore Burden of Disease Study.
(DOCX)

## Abbreviations

ANOVA: Analysis of Variance
ARPB: Average Relative Parameter Bias
CAT: Computerised Adaptive Testing
CFA: Confirmatory factor analysis
CFI: Comparative Fit Index
DIF: Differential Item Functioning
ECV: Explained Common Variance
EFA: Explantory Factor Analysis
GRM: Graded Response Model
HRQoL: Health-related Quality of Life
IECV: Item Explained Common Variance
OmegaH: Omega Hierarchical
IRT: Item Response Theory
PM: Positive Mindset
PMHI: Positive Mental Health Instrument
PROMIS: Patient Reported Outcome Measurement Information System
QIR: Qualitative Item Review
RMSEA: Root Mean Square Error of Approximation
SF-36: Short Form-36
SMWEB: Singapore Mental Wellbeing
TLI: Tucker Lewis Index
WHO: World Health Organization
WHOQOL-BREF: World Health Organization Quality of Life Scale

## References

[1] Kuhn S, Rieger UM. Health is a state of complete physical, mental and social well-being and not merely absence of disease or infirmity. Surgery for obesity and related diseases: official journal of the American Society for Bariatric Surgery. 2017;13(5):887 Epub 2017/04/09. 10.1016/j.soard.2017.01.046 . 28389194

[2] Thumboo J, Ow MYL, Uy EJB, Xin X, Chan ZYC, Sung SC, et al Developing a comprehensive, culturally sensitive conceptual framework of health domains in Singapore. PloS one. 2018;13(6):e0199881 Epub 2018/06/29. 10.1371/journal.pone.0199881 29953526 PMC6023157

[3] Vaingankar JA, Abdin E, Chong SA, Sambasivam R, Jeyagurunathan A, Seow E, et al Psychometric properties of the positive mental health instrument among people with mental disorders: a cross-sectional study. Health and quality of life outcomes. 2016;14:19 10.1186/s12955-016-0424-8 PubMed PMID: PMC4751680. 26868835 PMC4751680

[4] Fen CM, Isa I, Chu CW, Ling C, Ling SY. Development and validation of a mental wellbeing scale in Singapore. Psychology. 2013;4(07):592.

[5] Yost KJ, Waller NG, Lee MK, Vincent A. The PROMIS fatigue item bank has good measurement properties in patients with fibromyalgia and severe fatigue. Quality of life research: an international journal of quality of life aspects of treatment, care and rehabilitation. 2017;26(6):1417–26. Epub 2017/02/01. 10.1007/s11136-017-1501-0 . 28138862

[6] Victorson D, Tulsky DS, Kisala PA, Kalpakjian CZ, Weiland B, Choi SW. Measuring resilience after spinal cord injury: Development, validation and psychometric characteristics of the SCI-QOL Resilience item bank and short form. The journal of spinal cord medicine. 2015;38(3):366–76. Epub 2015/05/27. 10.1179/2045772315Y.0000000016 26010971 PMC4445027

[7] Batterham PJ, Sunderland M, Carragher N, Calear AL. Psychometric Properties of 7- and 30-Day Versions of the PROMIS Emotional Distress Item Banks in an Australian Adult Sample. Assessment. 2017:1073191116685809. Epub 2017/01/06. 10.1177/1073191116685809 . 28052687

[8] Boehm JK, Kubzansky LD. The heart's content: the association between positive psychological well-being and cardiovascular health. Psychological bulletin. 2012;138(4):655–91. Epub 2012/04/18. 10.1037/a0027448 . 22506752

[9] Steel Z, Marnane C, Iranpour C, Chey T, Jackson JW, Patel V, et al The global prevalence of common mental disorders: a systematic review and meta-analysis 1980–2013. International journal of epidemiology. 2014;43(2):476–93. Epub 2014/03/22. 10.1093/ije/dyu038 24648481 PMC3997379

[10] Uy EJB, Bautista DC, Xin X, Cheung YB, Thio ST, Thumboo J. Using best-worst scaling choice experiments to elicit the most important domains of health for health-related quality of life in Singapore. PloS one. 2018;13(2):e0189687 Epub 2018/02/09. 10.1371/journal.pone.0189687 29420564 PMC5805165

[11] Uy EJ, Xiao Y, Xin X, Yeo PTJ, Pua Y-H, Lee GL, et al. Developing Item Banks to Measure 3 Important Domains of Health-Related Quality of Life (HRQoL) in Singapore. 2018. 10.1186/s12955-019-1255-1 PMC6941315 31898541

[12] Kwan YH, Uy EJ, Bautista DC, Xin X, Xiao Y, Lee GL, et al Development and calibration of a novel social relationship item bank to measure health-related quality of life (HRQoL) in Singapore. Health and quality of life outcomes. 2019;17(1):82 10.1186/s12955-019-1150-9 31068201 PMC6505203

[13] Cella D, Yount S, Rothrock N, Gershon R, Cook K, Reeve B, et al The Patient-Reported Outcomes Measurement Information System (PROMIS): Progress of an NIH Roadmap Cooperative Group During its First Two Years. Medical care. 2007;45(5 Suppl 1):S3–S11. 10.1097/01.mlr.0000258615.42478.55 PubMed PMID: PMC2829758. 17443116 PMC2829758

[14] Health SMo. Singapore Burden of disease study 2010. 2014.

[15] Industry SMoT. Census of population 2010. 2011.

[16] Health SMo. National Health Survey 2004. 2005.

[17] Health SMo. National Health Survey 2007. 2009.

[18] (CASRO) CoASRO. On the Definition of Response Rates. 1982.

[19] Thumboo J, Ow MY, Uy EJB, Xin X, Chan ZYC, Sung SC, et al. Developing a comprehensive, culturally sensitive conceptual framework of health domains in Singapore. 2018. 10.1371/journal.pone.0199881 PMC6023157 29953526

[20] Statistics SDo. Census of Population 2010: Demographic Characteristics, Education, Language and Religion. In: Statistics SDo, editor. Singapore 2010.

[21] DM D. Bifactor Indices Calculator: A Microsoft Excel-based tool to calculate various indices relevant to bifactor CFA models 2016. Available from: http://sites.education.uky.edu/apslab/resources.

[22] L.K. M, B.O. M. Mplus User's Guide. Sixth ed. Los Angeles, CA2010.

[23] Steven PR, Richard S, Keith FW, Mark GH. Multidimensionality and Structural Coefficient Bias in Structural Equation Modeling: A Bifactor Perspective. Educational and Psychological Measurement. 2012;73(1):5–26. 10.1177/0013164412449831

[24] PROMIS Instrument Development and Validation Scientific Standards Version 2.0. 2013.

[25] Reise SP, Bonifay WE, Haviland MG. Scoring and modeling psychological measures in the presence of multidimensionality. Journal of personality assessment. 2013;95(2):129–40. Epub 2012/10/04. 10.1080/00223891.2012.725437 . 23030794

[26] Amtmann D, Cook KF, Jensen MP, Chen WH, Choi S, Revicki D, et al Development of a PROMIS item bank to measure pain interference. Pain. 2010;150(1):173–82. Epub 2010/06/18. 10.1016/j.pain.2010.04.025 20554116 PMC2916053

[27] Brian DS, David T, Maria Orlando E. Using Logistic Approximations of Marginal Trace Lines to Develop Short Assessments. Applied Psychological Measurement. 2012;37(1):41–57. 10.1177/0146621612462759

[28] Brian DS, Maria Orlando E. Using Hierarchical IRT Models to Create Unidimensional Measures From Multidimensional Data Handbook of Item Response Theory Modeling: Routledge; 2014.

[29] Schellekens MPJ, Tamagawa R, Labelle LE, Speca M, Stephen J, Drysdale E, et al Mindfulness-Based Cancer Recovery (MBCR) versus Supportive Expressive Group Therapy (SET) for distressed breast cancer survivors: evaluating mindfulness and social support as mediators. Journal of behavioral medicine. 2017;40(3):414–22. Epub 2016/10/11. 10.1007/s10865-016-9799-6 27722908 PMC5406481

[30] Youssef CM, Luthans F. Positive Organizational Behavior in the Workplace:The Impact of Hope, Optimism, and Resilience. Journal of Management. 2007;33(5):774–800. 10.1177/0149206307305562

[31] Harolds JA. Quality and Safety in Healthcare, Part XLVII: Resilience and Burnout. Clinical nuclear medicine. 2019;44(5):394–6. Epub 2018/10/10. 10.1097/RLU.0000000000002303 . 30300205

[32] Crins MHP, Terwee CB, Klausch T, Smits N, de Vet HCW, Westhovens R, et al The Dutch-Flemish PROMIS Physical Function item bank exhibited strong psychometric properties in patients with chronic pain. Journal of clinical epidemiology. 2017;87:47–58. Epub 2017/04/02. 10.1016/j.jclinepi.2017.03.011 . 28363734

[33] Haley SM, Fragala-Pinkham MA, Dumas HM, Ni P, Gorton GE, Watson K, et al Evaluation of an item bank for a computerized adaptive test of activity in children with cerebral palsy. Physical therapy. 2009;89(6):589–600. Epub 2009/05/09. 10.2522/ptj.20090007 19423642 PMC2689784

[34] Zamanzadeh V, Ghahramanian A, Rassouli M, Abbaszadeh A, Alavi-Majd H, Nikanfar A-R. Design and Implementation Content Validity Study: Development of an instrument for measuring Patient-Centered Communication. Journal of Caring Sciences. 2015;4(2):165–78. 10.15171/jcs.2015.017 PubMed PMID: PMC4484991. 26161370 PMC4484991

[35] Kern ML, Waters LE, Adler A, White MA. A multidimensional approach to measuring well-being in students: Application of the PERMA framework. The journal of positive psychology. 2015;10(3):262–71. Epub 2015/03/10. 10.1080/17439760.2014.936962 25745508 PMC4337659

[36] Sow WT, Wee HL, Wu Y, Tai ES, Gandek B, Lee J, et al Normative Data for the Singapore English and Chinese SF-36 Version 2 Health Survey. Annals of the Academy of Medicine, Singapore. 2014;43(1):15–23. Epub 2014/02/22. . 24557461

[37] Muthén B, Kaplan D, Hollis M. On structural equation modeling with data that are not missing completely at random. Psychometrika. 1987;52(3):431–62. 10.1007/BF02294365

