# Supplementary material for: Development and calibration of a novel positive mindset item bank to measure health-related quality of life (HRQoL) in Singapore
Source: PLoS One. 2019 Jul 31;14(7):e0220293. doi: 10.1371/journal.pone.0220293 (PMC6668803; doi:10.1371/journal.pone.0220293)
Supplement: S1 Table — Based on the 2010 Singapore Burden of Disease Study. (DOCX) [file pone.0220293.s001.docx]

**S1 Table.** Chronic Illnesses qualifying for patient recruitment

| **List A** | **List B** |
| --- | --- |
| - Age-related macular degeneration - Anxiety disorder - Asthma - Breast cancer - Chronic obstructive pulmonary disease (COPD) - Colon cancer - Depression - Diabetes - Diabetic retinopathy - Glaucoma - Hearing loss, adult-onset - Heart failure - Ischemic heart disease - Lung cancer - Migraine - Osteoarthritis - Rheumatoid arthritis - Stroke | - Cancer except breast or color cancer - Epilepsy - Heart disease except heart failure, ischemic heart disease - High cholesterol - Hypertension - Joint disease except rheumatoid arthritis or osteoarthritis - Kidney disease not requiring dialysis or transplantation - Benign prostate hyperplasia - Osteoporosis |

Based on the 2010 Singapore Burden of Disease Study
